# Discordance Between Glucose Levels Measured in Interstitial Fluid *vs* in Venous Plasma After Oral Glucose Administration: A *Post-Hoc* Analysis From the Randomised Controlled PRE-D Trial

**DOI:** 10.3389/fendo.2021.753810

**Published:** 2021-10-05

**Authors:** Kristine Færch, Hanan Amadid, Lea Bruhn, Kim Katrine Bjerring Clemmensen, Adam Hulman, Mathias Ried-Larsen, Martin Bæk Blond, Marit Eika Jørgensen, Dorte Vistisen

**Affiliations:** ^1^ Steno Diabetes Center Copenhagen, Gentofte, Denmark; ^2^ Department of Biomedical Sciences, University of Copenhagen, Copenhagen, Denmark; ^3^ Steno Diabetes Center Aarhus, Aarhus, Denmark; ^4^ Centre for Physical Activity Research, Rigshospitalet, Copenhagen, Denmark; ^5^ Institute of Sports and Clinical Biomechanics, University of Southern Denmark, Odense, Denmark; ^6^ University of Southern Denmark, Copenhagen, Denmark; ^7^ Department of Public Health, University of Copenhagen, Copenhagen, Denmark

**Keywords:** oral glucose challenge test, continuous glucose monitor system, prediabetes, Bland-Altman, proportional bias

## Abstract

**Aims:**

The oral glucose tolerance test (OGTT) is together with haemoglobin A_1c_ (HbA_1c_) gold standard for diagnosing prediabetes and diabetes. The objective of this study was to assess the concordance between glucose values obtained from venous plasma versus interstitial fluid after oral glucose administration in 120 individuals with prediabetes and overweight/obesity.

**Methods:**

120 adults with prediabetes defined by HbA_1c_ 39-47 mmol/mol and overweight or obesity who participated in the randomised controlled PRE-D trial were included in the study. Venous plasma glucose concentrations were measured at 0, 30, 60 and 120 minutes during a 75 g oral glucose tolerance test (OGTT) performed on three different occasions within a 26 weeks period. During the OGTT, the participants wore a CGM device (IPro2, Medtronic), which assessed glucose concentrations every five minutes.

**Results:**

A total of 306 OGTTs with simultaneous CGM measurements were obtained. Except in fasting, the CGM glucose values were below the OGTT values throughout the OGTT period with mean (SD) differences of 0.2 (0.7) mmol/L at time 0 min, -1.1 (1.3) at 30 min, -1.4 (1.8) at 60 min, and -0.5 (1.1) at 120 min). For measurements at 0 and 120 min, there was a proportional bias with an increasing mean difference between CGM and OGTT values with increasing mean of the two measurements.

**Conclusions:**

Due to poor agreement between the OGTT and CGM with wide 95% limits of agreement and proportional bias at 0 and 120 min, the potential for assessing glucose tolerance in prediabetes using CGM is questionable.

## Introduction

The oral glucose tolerance test (OGTT) provides important information about fasting and post-challenge glucose metabolism and is together with haemoglobin A_1c_ (HbA_1c_) gold standard for diagnosing prediabetes and diabetes (1). However, because the OGTT is inconvenient and time consuming it is seldom used in clinical practice. The use of HbA_1c_ for diagnosing diabetes and especially prediabetes is also challenging, as HbA_1c_ levels in the non-diabetic range is affected by several factors not related to glycaemia (e.g. genetics, iron-deficiency, anaemia, etc.) (2, 3).

In recent years, continuous glucose monitoring (CGM) has become widely used for clinical purposes, because it replaces self-monitoring of glucose among diabetes patients and gives detailed information on glucose excursions during free-living conditions. As such, glucose concentrations measured by a glucose sensor (CGM) placed in the subcutaneous tissue for several days may be more physiologically and clinically relevant for assessing glucose tolerance than a single OGTT. Glycaemic variability assessed by the CGM is associated with the development of diabetic complications even in people with well-controlled HbA_1c_ levels (4), which makes the CGM relevant as a monitor of cardiometabolic risk.

Because glucose concentrations during an OGTT are measured in venous blood and glucose concentrations using CGMs are measured in the interstitial fluid, differences in glucose concentrations between the two methods are expected, but knowledge on the magnitude of the difference and the time-lag between the measures are still limited, especially among people without diabetes. Studies have found the time-lag in glucose readings from CGMs compared to plasma glucose concentrations to be of approx. 5-10 min during hyperglycaemic excursions using data from 14 people with type 1 diabetes (5, 6). In another study of 15 healthy individuals subjected to OGTTs, the time-lag was on average 15 min (7). Also, using model simulations, it has been suggested that CGMs overestimate low glucose values, but underestimate high glucose values, leading to an underestimation of both hypo- and hyperglycaemic events in people with diabetes (6). In healthy non-diabetic individuals, CGMs also seem to underestimate plasma glucose levels during hyper-insulinemic conditions (8). Studies of the relationship between interstitial and plasma glucose concentrations during glucose stimulation in individuals with prediabetes are lacking. Thus, the objective of this study was to assess the concordance between glucose values obtained from venous plasma versus interstitial fluid after oral glucose administration in 120 individuals with prediabetes and overweight/obesity who had three repeated measures over 6 months. Specifically, we examined: 1) the time-lag in interstitial glucose compared with blood glucose during an OGTT and 2) the concordance between blood and interstitial glucose concentrations after taking the time-lag into account.

## Methods

### Participants and Setting

We used data from a randomised, multi-arm, parallel, controlled trial, the PRE-D Trial (9, 10). Between February 2016 and July 2019, 120 men and women with BMI ≥25 and HbA_1c_ levels in the prediabetic range (5.7-6.4%/39-47 mmol/mol) were randomised to one of four interventions for 13 weeks: 1) dapagliflozin (10 mg once daily); 2) metformin (850 mg twice daily); 3) exercise (interval training, 30 min, 5 times per week); or 4) control (habitual living). The 13 weeks of intervention were followed by another period of 13 weeks where no interventions were provided. The PRE-D Trial is described in detail elsewhere (9, 10). The study was conducted in accordance with the Helsinki II declaration and Good Clinical Practice. The protocol was approved by the Ethics Committee of the Capital Region (H-15011398) and the Danish Medicines Agency (EudraCT number: 2015-001552-30). Approval for data storage was obtained from the Danish Data Protection Board (2012-58-0004). All participants provided written informed consent before taking part in the study.

### Examinations

At baseline and at 13 and 26 weeks, the participants attended the research facility at Steno Diabetes Center Copenhagen, Gentofte, Denmark, for a clinical examination after an overnight fast of ≥8 hours. Upon arrival between 08:00-9:00 AM on the day of the clinical examination, the participants had a CGM attached (iPro2 CGM with Enlite sensor, Medtronic Denmark A/S, Copenhagen, Denmark), which was used to assess glucose concentrations every five minutes during the following six days. The CGM was calibrated after one and two hours. After the second calibration, 75 g glucose was administered orally and venous blood samples for assessment of plasma glucose concentrations were drawn at 0, 30, 60, and 120 min. Questionnaires on socio-economic factors, health, and disease were filled in during the OGTT. During the test day, measurements of height, body weight, waist and hip circumference, and blood pressure were also performed, and body composition was measured by Dual-Energy X-ray Absorptiometry (Discovery DXA System, Hologic, Marlborough, Massachusetts, USA).

Following each test day (baseline, 13 weeks, 26 weeks), interstitial glucose levels were monitored for six consecutive days during free-living with the CGM system. The CGM provided glucose measurements every 5 min during the entire measurement period (both during the OGTT and free living). To calibrate the CGMs, the participants measured blood glucose levels at home four times a day (before breakfast, before lunch, before dinner, and before bedtime) for the following 6 days using a glucometer (Contour XT, Ascensia Diabetes Care Denmark ApS, Copenhagen, Denmark).

### Biochemical Analysis

Samples for biochemical analysis of plasma glucose concentrations were put on ice immediately following sampling. Samples were centrifuged shortly after collection at 4000 rpm for 15 minutes (Sigma 4K15, Osterode Am Harz, Germany), except for samples used for analysis of HbA_1c_ and serum insulin concentrations. Samples for analysis of serum insulin concentration were centrifuged 30 minutes after collection. The samples were stored in a refrigerator for the remainder of the test day. Serum insulin was analysed using electro-chemiluminescence immunoassay (Cobas e411, Roche Diagnostics, Switzerland). HbA_1c_ was measured by High Performance Liquid Chromatography (Tosoh G8, Tosoh Corporation, Japan). Plasma glucose, total cholesterol, HDL cholesterol, and triglycerides were analysed by cholometric analysis (Vitros 5600, Ortho Clinical Diagnostics, USA). Plasma VLDL cholesterol was calculated as plasma triglycerides (mmol/l) divided by 2.2, and plasma LDL cholesterol was calculated based on the Friedewald equation (11). Estimated glomerular filtration rate (eGFR) was calculated using the CKD-epi formula (12).

### Data Management, Calculations and Definitions

Raw data from the CGM and glucometer were downloaded from the online system CareLink™ (Medtronic MiniMed, Northridge, CA, USA). The mean amplitude of glycaemic excursions (MAGE), a measure of glycaemic variability, was calculated by taking the arithmetic mean of the blood glucose increases or decreases when both ascending and descending segments exceeded the value of one standard deviation of the blood glucose during a 24-hour measurement period. Data from the free-living measurement period was used for calculating MAGE.

### Statistical Analysis

**Figure 1:**
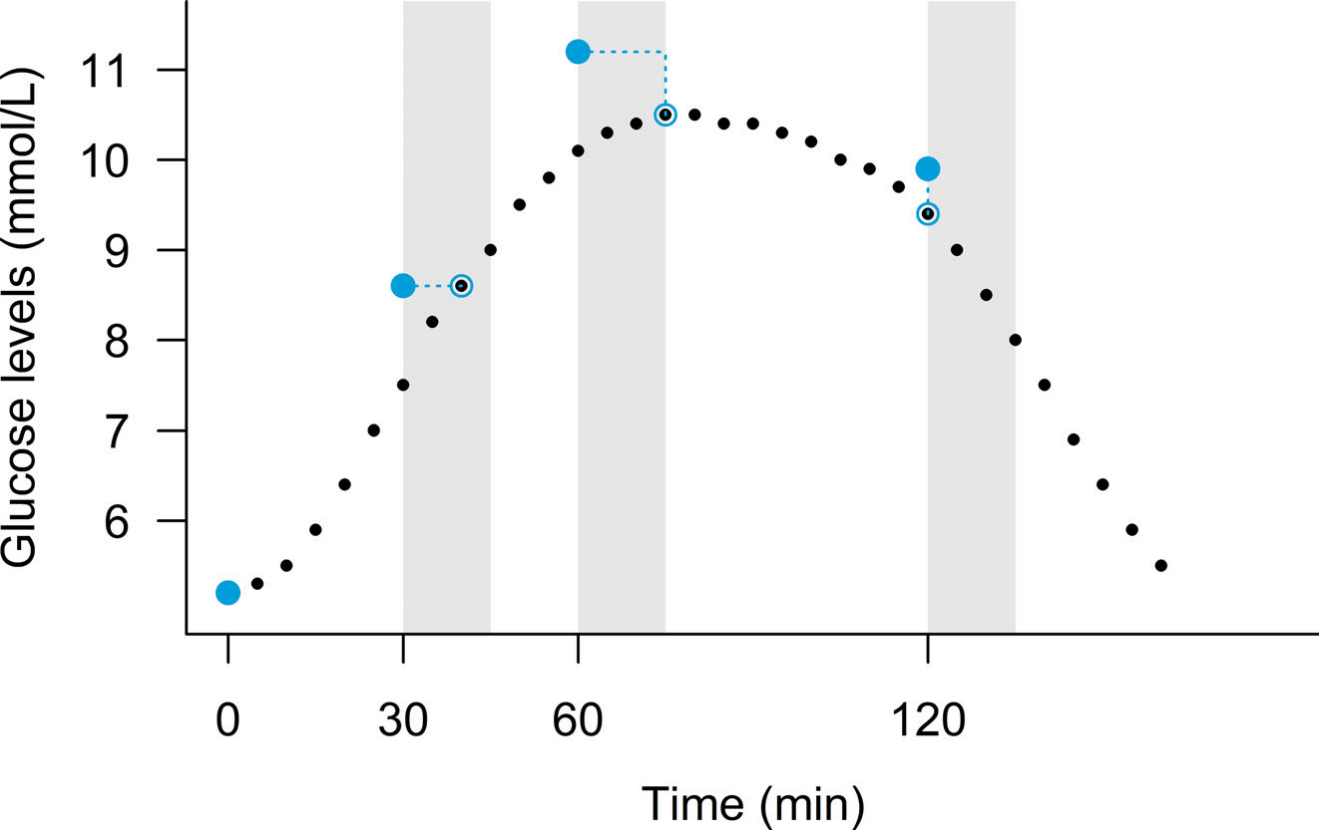
An example of (plasma glucose levels) measured during the OGTT (large blue points) and simultaneously by the continuous glucose monitoring device (black points) in a person. The light grey areas indicate the 0-15 min period after the OGTT measurements, and the dotted blue lines point to the best CGM match within the 15 min period.

Linear mixed-effects analysis with a participant-specific random intercept was used to estimate population mean levels of plasma glucose at each CGM and OGTT time-point. For all participants, we matched each of their post-challenge OGTT values to their least deviating CGM value in the period 0-15 min after the OGTT sample was obtained. In **Figure 1**, this method is illustrated for one of the participants. Because the CGM provided glucose measurements every 5 min, the time of best match was at 0, 5, 10 or 15 min after the OGTT sample. For the best matching CGM value, the corresponding time-lag and observed difference in glucose level between the CGM and OGTT was recorded.

To examine the agreement between glucose concentrations obtained from subcutaneous tissue versus venous plasma, Bland-Altman plots (13) were performed for each time point during the OGTT using the best matching CGM value. Potential heteroscedasticity was assessed graphically. We estimated limits of agreement and tested for proportional bias using linear mixed-effects analysis with a participant-specific random intercept.

We assumed that the association between glucose levels measured by the OGTT and CGM was unaffected by the interventions. However, in a sensitivity analysis, we studied the potential confounding effect of the different interventions on the relationship between CGM and OGTT glucose data by repeating all analyses including only data from the baseline visit.

Statistical analyses were performed in R version 3.5.2 (The R Foundation for Statistical Computing).

## Results

### Clinical Characteristics

The median (Q1; Q3) age of the study population was 63 (54; 68) years, BMI was 30.8 (27.4; 34.3) kg/m^2^, and 44% were men. A total of 306 OGTTs with simultaneous valid CGM measurements were obtained. In **Table 1**, the characteristics of the study participants are shown.

**Table 1:** Characteristics of study participants at baseline (n = 120).

Age (years)	62.6 (54.0,68.0)
Men (n, %)	53 (44)
Current smoker (n, %)	13 (11)
Family history of diabetes (n, %)	64 (53)
Family history of CVD (n, %)	70 (58)
Antihypertensive medication (n, %)	32 (27)
Lipid-lowering medication (n, %)	28 (23)
Systolic blood pressure (mmHg)	131 (122,144)
Diastolic blood pressure (mmHg)	85 (79,90)
Body weight (kg)	91 (82,104)
BMI (kg/m^2^)	30.8 (27.4,34.3)
Waist circumference (cm)	104 (98,113)
Body fat (%)	40 (31,44)
eGFR (ml/min/1.73 m^2^)	88.5 (80.6,97.5)
Total cholesterol (mmol/l)	5.1 (4.3,5.9)
LDL cholesterol (mmol/l)	3.1 (2.4,3.7)
HDL cholesterol (mmol/l)	1.3 (1.1,1.5)
Triglycerides (mmol/l)	1.3 (0.9,1.8)
HbA_1c_	
mmol/mol	41 (39,43)
%	5.9 (5.7,6.1)
Fasting plasma glucose (mmol/l)	5.6 (5.2,5.9)
Fasting serum insulin (pmol/l)	71.5 (48.0,98.5)
MAGE (mmol/l)	1.6 (1.4,2.2)

Data are medians (Q1;Q3) or numbers (%).

### Time-Lag Between CGM and OGTT

**Figure 2:**
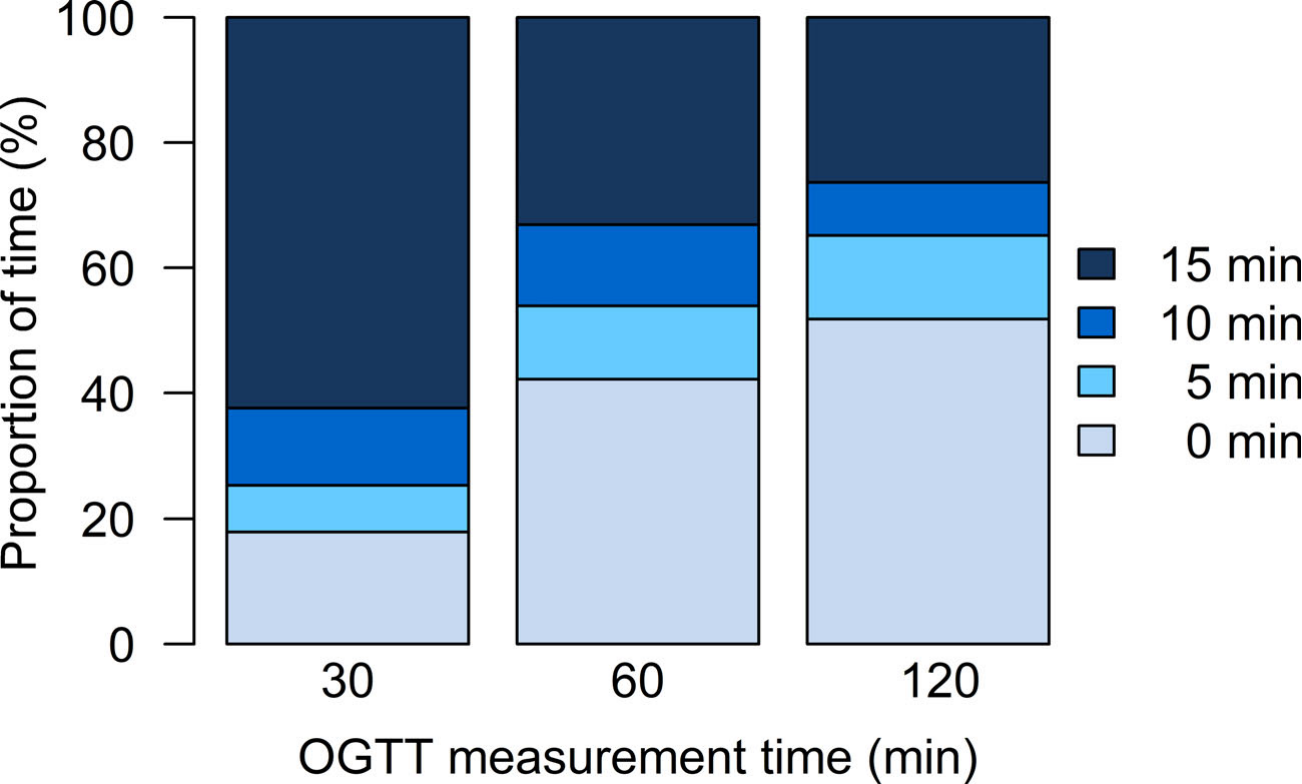
Distribution of possible time-lags (0, 5, 10 or 15 min) of the corresponding CGM measurement for each of the post-challenge OGTT glucose values.

**Figure 2** shows the distribution of possible time-lags (0, 5, 10 or 15 min) of the corresponding CGM measurement for each of the post-challenge OGTT glucose values. For the OGTT value at 30 min, there was a 15 min lag-time in CGM measurements for more than 60% of the participants. For the OGTT values at 60 and 120 min, the best match of CGM measurements was at the same time as the OGTT measurements for approximately half of the participants, but in 33% and 26% of the participants, a 15 min lag-time in CGM measurements at 60 and 120 min, respectively, was present.

### Concordance Between Blood and Interstitial Glucose Concentrations

**Figure 3:**
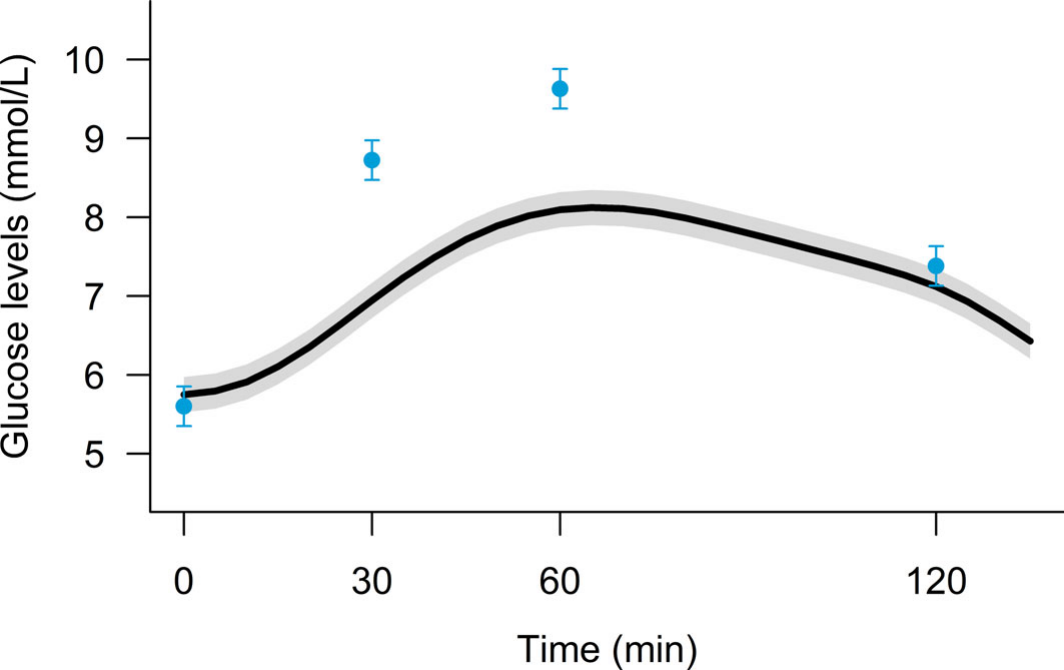
Mean (95%-CI) plasma glucose levels measured during the OGTT (blue points) and simultaneously by the continuous glucose monitoring device (black curve) in 120 persons with prediabetes examined three times over 26 weeks.

**Figure 3** illustrates the population mean plasma glucose levels measured during the OGTT and simultaneously by the CGM device. Except in the fasting state, the mean CGM glucose values were on average below the mean OGTT values throughout the 120 min period, and this was especially pronounced at 60 min. The mean (SD) differences between observed CGM and OGTT glucose concentrations were 0.2 (0.7) mmol/L (equivalent to 3.2 (13.4)%) at time 0 min, -1.1 (1.3) mmol/L (-12.2 (15.4)%) at 30 min, -1.4 (1.8) mmol/L (-13.3 (18.2)%) at 60 min, and -0.5 (1.1) mmol/L (-3.9 (14.9)%) at 120 min.

**Figure 4:**
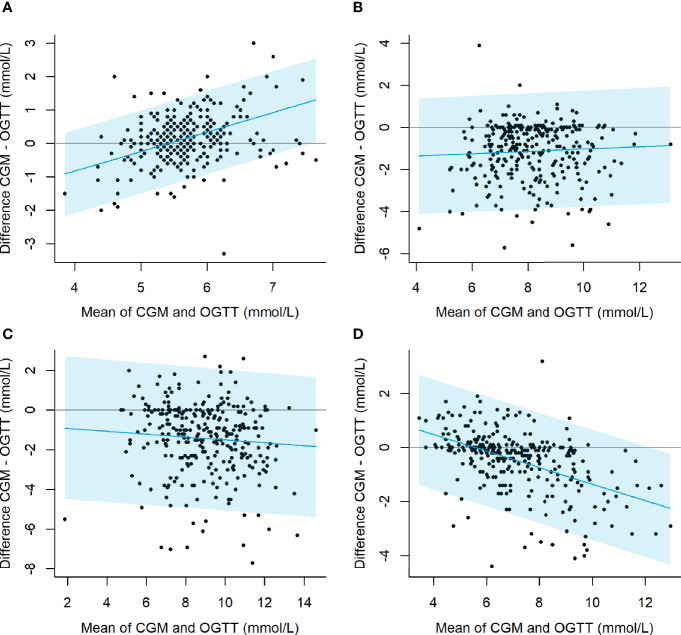
Bland-Altman plots illustrating the agreement between the CGM and OGTT glucose measured during fasting **(A)**, and after 30 min **(B)**, 60 min **(C)**, and 120 min **(D)** after oral administration of 75 g glucose. Light blue areas indicate limits of agreement. Test of proportional bias: 0 min: *P <*0.001, 0 min: *P* = 0.306, 0 min: *P* = 0.196, 120 min: *P <*0.001.

The Bland-Altman analyses at time points 0, 30, 60 and 120 min are presented in **Figure 4**. For the measurements at 0 and 120 min there was a proportional bias with an increasing or decreasing mean difference between CGM and OGTT values (y-axis) with increasing mean of the two measurements (x-axis) (0 min: *P <*0.001, 120 min: *P <*0.001). Hence, during fasting conditions, the CGM particularly overestimated glucose values for high mean values (slope 0.6 per mmol/L), and at 120 min the CGM greatly underestimated glucose at high mean values (slope -0.3 per mmol/L). There was no sign of heteroscedasticity with limits of agreement being overall parallel to the mean curve in any of the plots in **Figure 4**. The sensitivity analysis including only data from the baseline visit showed similar results (**Electronic Supplementary Material**).

## Discussion

The use of glucose sensors to inform diabetes management decisions has become part of most practices during recent years (14). In contrast, the potential usefulness of CGMs to guide diagnostic decisions has received less attention. In this analysis of 120 individuals with prediabetes and overweight or obesity, we show that glucose levels obtained by CGMs during an OGTT are on average 12-13% lower at 30 and 60 min and 4% lower at 120 min after oral glucose administration than those measured in venous plasma – even when taking individual time-lag in sensor glucose measurements into account.

Plasma and interstitial fluid are both part of the body’s extracellular fluid, and interstitial fluid can be considered the ultrafiltrate of plasma, which transports nutrients, including glucose, from the blood stream to the cells and back. Therefore, using glucose concentrations determined from CGMs with real-time feedback seems highly relevant in evaluation of glucose tolerance in individuals at a high risk for diabetes (15). However, there is a lack of studies with concomitant analysis of OGTT and CGM data. Previous studies on CGM accuracy compared to blood glucose concentrations have included people with diabetes during a liquid meal test (5), insulin-induced hypoglycaemic conditions (16), or during a 24-hour hospital stay (16) (not OGTT). Also, one study of 11 young healthy adults found 15% lower interstitial glucose concentrations than plasma glucose concentrations concomitantly measured during a stepped euglycemic-hypoglycaemic-hyperglycaemic insulin clamp (8). Another study in 15 healthy overweight men subjected to an OGTT found that the time to peak of glucose was significantly delayed for the interstitial fluid measurement compared to the plasma glucose measurement and that body fat percentage was related to the time to peak (7). Using Bland-Altman plots, the study also suggested that the differences between the plasma glucose and interstitial fluid measures increased with increasing level of circulating glucose (7), which is in alignment with our findings. Together, our findings and findings from other studies underscore that interstitial glucose concentrations do not sufficiently capture plasma glucose when glucose levels are acutely changed. This is not surprising as several factors contribute to the concentration difference and time-lag between glucose measured in the venous plasma and the interstitial fluid, including the rate of glucose diffusion, the magnitude of concentration differences in various tissues, blood flow, blood vessel permeability to glucose, and acute changes in the release of insulin and glucagon (17, 18). Also, the fact that the OGTT was performed within the first 24 hours of the CGM measurement period may have contributed to the limited agreement between the two measures, because the accuracy of the CGM may be lower on the first day of measurement.

A strength of this analysis was the availability of up to three pairwise measures of CGM and OGTT data for 120 participants within 26 weeks, enabling us to study the difference between venous plasma glucose and interstitial glucose during a dynamic but standardised change of glucose concentration. The different interventions are not likely to affect the association between glucose measured in venous plasma and in the interstitial fluid, and our sensitivity analysis showed that analysis of baseline data produced comparable results. A limitation of our study was that we only studied individuals with prediabetes and overweight or obesity. Accordingly, we were not able to test the potential of the CGM to distinguish between individuals with normoglycaemia and prediabetes – an aspect which has been addressed in previous studies with emphasis on the role of glycaemic variability (19–21). Another limitation is related to the type of CGM used in this study. Our findings may be specific to the iPro2 sensor and may not be generalised to other types of sensors, for instance the FreeStyle Libre Flash CGM system by Abbott, which is now more commonly used in clinical settings. The FreeStyle Libre Flash only assesses glucose concentrations every 15 min as compared to the IPro2 where glucose concentrations are assessed every 5 min, which is an additional challenge. Therefore, more studies using different types of CGMs together with the OGTT are warranted.

Not unexpectedly the CGM systematically underestimated the glucose level when compared to plasma samples with 13% discrepancy between observed OGTT and CGM levels. In general, there is a high intra-individual variation in fasting glucose (15%) and 2-hour glucose (46%) concentrations during an OGTT (22). We also found a large interindividual variation in the difference between the results for the two methods. However, more critically we found a proportional bias in the difference between OGTT and CGM levels and inter-individual differences in the time-lag, making it unlikely that the IPro2 can be used as a substitute for plasma samples when performing an OGTT. As such, crude CGM measures may not be accurate enough to assess glucose tolerance among individuals with prediabetes. Further investigations are needed to assess the link between CGM measures and long-term outcomes before CGMs can be used for diagnostic purposes.

## Data Availability Statement

Data described in the article and analytic code will be made available upon request to the corresponding author pending application and approval.

## Ethics Statement

The studies involving human participants were reviewed and approved by The Ethics Committee of the Capital Region, Region Hovedstaden, Blegdamsvej 60. 1. sal, 2100 København Ø. The patients/participants provided their written informed consent to participate in this study.

## Author Contributions

KF and DV conceived the idea and drafted the manuscript. KF is the sponsor and MJ is the principal investigator of the PRE-D Trial. HA, LB, and MR-L contributed to the design of the study. KF, HA, LB, KC, MB, and MJ were involved in the conduct of the trial and data collection. KC and DV performed statistical analyses. AH, MR-L, and MB provided statistical input. All authors critically revised the manuscript for important intellectual content and approved the final version of the manuscript. KF is the guarantor of this work and, as such, had full access to all the data in the study, takes responsibility for the integrity of the data and the accuracy of the data analysis, and had final responsibility for the decision to submit for publication. All authors contributed to the article and approved the submitted version.

## Funding

The study was funded by the Novo Nordisk Foundation, AstraZeneca AB, the Danish Innovation Foundation, and University of Copenhagen. The funders had no role in study design, data collection, data analysis, interpretation, or writing of the report. The corresponding author had full access to all data in the study and had final responsibility for the decision to submit for publication.

## Conflict of Interest

KF, HA, LB, KC, MB, MJ, and DV are employed by Steno Diabetes Center Copenhagen, a research hospital working in the Danish National Health Service. Until 31 December 2016, Steno Diabetes Center was owned by Novo Nordisk A/S. KF, DV, KC, and MJ own shares in Novo Nordisk A/S. KF has received research support from AstraZeneca and Unilever, is member of the Board of Directors for ChemoMetec A/S. MR-L has received personal lecture fees from Novo Nordisk A/S. MJ has received research grants from Amgen, Sanofi Aventis, Boehringer Ingelheim, and Astra Zeneca. DV has received research grants from Boehringer Ingelheim and Bayer A/S.

The remaining author declares that the research was conducted in the absence of any commercial or financial relationships that could be construed as a potential conflict of interest.

## Publisher’s Note

All claims expressed in this article are solely those of the authors and do not necessarily represent those of their affiliated organizations, or those of the publisher, the editors and the reviewers. Any product that may be evaluated in this article, or claim that may be made by its manufacturer, is not guaranteed or endorsed by the publisher.
