# Supplementary material for: Discordance Between Glucose Levels Measured in Interstitial Fluid vs in Venous Plasma After Oral Glucose Administration: A Post-Hoc Analysis From the Randomised Controlled PRE-D Trial
Source: Front Endocrinol (Lausanne). 2021 Oct 5;12:753810. doi: 10.3389/fendo.2021.753810 (PMC8525890; doi:10.3389/fendo.2021.753810)
Supplement: Supplementary file 1 [file DataSheet_1.docx]

**Electronic Supplementary Material**

**
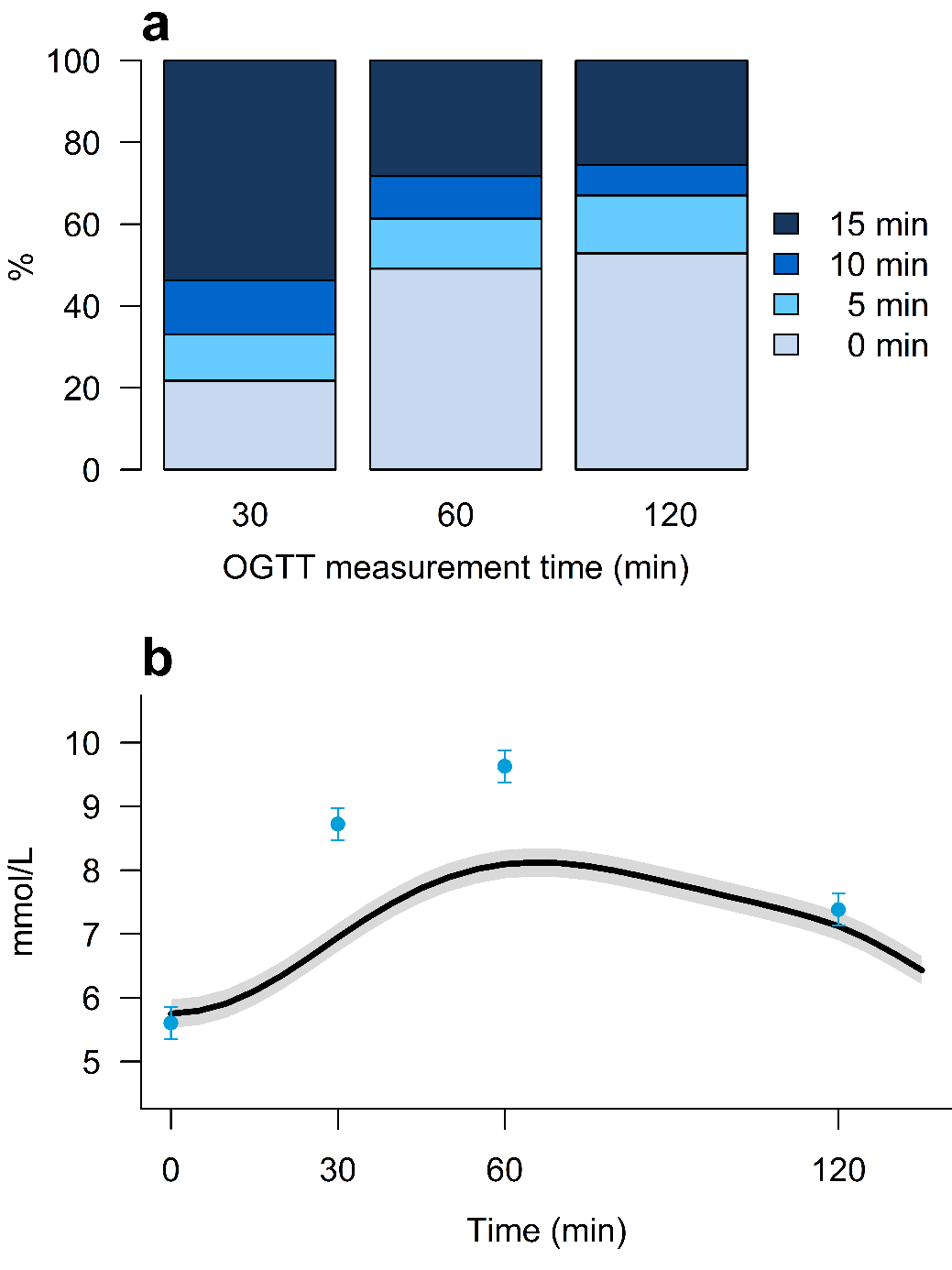
**

**ESM Figure 1:** Panel a: Distribution of times of best match of CGM data with the post-challenge OGTT glucose values. Panel b: Mean (95%-CI) plasma glucose levels measured during the OGTT (blue points) and simultaneously by the continuous glucose monitoring device (black curve) in 120 persons with prediabetes examined three times over 26 weeks. Analyses are based on baseline data only (sensitivity analysis). The mean (SD) differences between observed CGM and OGTT glucose concentrations were 0.1 (0.7) mmol/L at time 0 min, -1.1 (1.3) mmol/L at 30 min, -1.5 (1.9) mmol/L at 60 min, and -0.6 (1.2) mmol/L at 120 min.


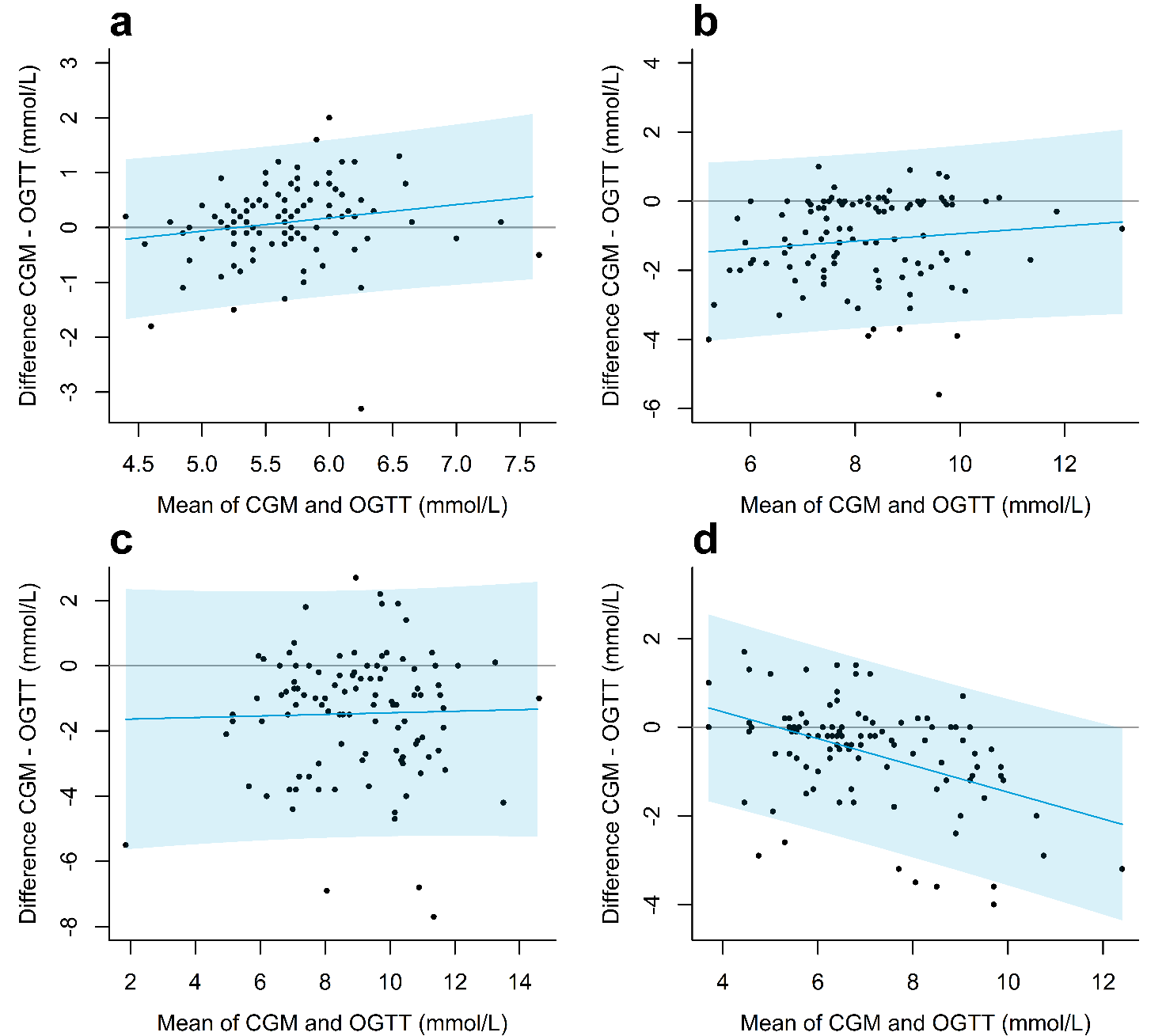


**ESM Figure 2:** Bland-Altman plots illustrating the agreement between the CGM and OGTT glucose measured during fasting (a), and after 30 min (b), 60 min (c), and 120 min (d) after oral administration of 75 g glucose. Light blue area indicates limits of agreement. Analyses are based on baseline data only *(sensitivity analysis)*. Test of proportional bias: 0 min: *P* = 0.064, 0 min: *P* = 0.215, 0 min: *P* = 0.791, 120 min: *P* <0.001
